# Association between particulate air pollution and hypertensive disorders in pregnancy: A retrospective cohort study

**DOI:** 10.1371/journal.pmed.1004395

**Published:** 2024-04-26

**Authors:** Yi Sun, Rashmi Bhuyan, Anqi Jiao, Chantal C. Avila, Vicki Y. Chiu, Jeff M. Slezak, David A. Sacks, John Molitor, Tarik Benmarhnia, Jiu-Chiuan Chen, Darios Getahun, Jun Wu

**Affiliations:** 1 Institute of Medical Information, Chinese Academy of Medical Sciences and Peking Union Medical College, Beijing, China; 2 Department of Environmental and Occupational Health, Program in Public Health, University of California, Irvine, California, United States of America; 3 Occupational and Environmental Medicine Residency Program, University of California, Irvine, California, United States of America; 4 Department of Occupational Medicine, Kaiser Permanente Northern California, Antioch, California, United States of America; 5 Department of Research & Evaluation, Kaiser Permanente Southern California, Pasadena, California, United States of America; 6 Department of Obstetrics and Gynecology, University of Southern California, Keck School of Medicine, Los Angeles, California, United States of America; 7 College of Public Health and Human Sciences, Oregon State University, Corvallis, Oregon, United States of America; 8 Scripps Institution of Oceanography, University of California, San Diego, California, United States of America; 9 Departments of Population & Public Health Sciences and Neurology, University of Southern California, Keck School of Medicine, Los Angeles, California, United States of America; 10 Department of Health Systems Science, Kaiser Permanente Bernard J. Tyson School of Medicine, Pasadena, California, United States of America; The University of Edinburgh Usher Institute of Population Health Sciences and Informatics, UNITED KINGDOM

## Abstract

**Background:**

Epidemiological findings regarding the association of particulate matter ≤2.5 μm (PM_2.5_) exposure with hypertensive disorders in pregnancy (HDP) are inconsistent; evidence for HDP risk related to PM_2.5_ components, mixture effects, and windows of susceptibility is limited. We aimed to investigate the relationships between HDP and exposure to PM_2.5_ during pregnancy.

**Methods and findings:**

A large retrospective cohort study was conducted among mothers with singleton pregnancies in Kaiser Permanente Southern California from 2008 to 2017. HDP were defined by International Classification of Diseases-9/10 (ICD-9/10) diagnostic codes and were classified into 2 subcategories based on the severity of HDP: gestational hypertension (GH) and preeclampsia and eclampsia (PE-E). Monthly averages of PM_2.5_ total mass and its constituents (i.e., sulfate, nitrate, ammonium, organic matter, and black carbon) were estimated using outputs from a fine-resolution geoscience-derived model. Multilevel Cox proportional hazard models were used to fit single-pollutant models; quantile g-computation approach was applied to estimate the joint effect of PM_2.5_ constituents. The distributed lag model was applied to estimate the association between monthly PM_2.5_ exposure and HDP risk.

This study included 386,361 participants (30.3 ± 6.1 years) with 4.8% (17,977/373,905) GH and 5.0% (19,381/386,361) PE-E cases, respectively. In single-pollutant models, we observed increased relative risks for PE-E associated with exposures to PM_2.5_ total mass [adjusted hazard ratio (HR) per interquartile range: 1.07, 95% confidence interval (CI) [1.04, 1.10] *p* < 0.001], black carbon [HR = 1.12 (95% CI [1.08, 1.16] *p* < 0.001)] and organic matter [HR = 1.06 (95% CI [1.03, 1.09] *p* < 0.001)], but not for GH. The population attributable fraction for PE-E corresponding to the standards of the US Environmental Protection Agency (9 μg/m^3^) was 6.37%. In multi-pollutant models, the PM_2.5_ mixture was associated with an increased relative risk of PE-E ([HR = 1.05 (95% CI [1.03, 1.07] *p* < 0.001)], simultaneous increase in PM_2.5_ constituents of interest by a quartile) and PM_2.5_ black carbon gave the greatest contribution of the overall mixture effects (71%) among all individual constituents. The susceptible window is the late first trimester and second trimester. Furthermore, the risks of PE-E associated with PM_2.5_ exposure were significantly higher among Hispanic and African American mothers and mothers who live in low- to middle-income neighborhoods (*p* < 0.05 for Cochran’s Q test). Study limitations include potential exposure misclassification solely based on residential outdoor air pollution, misclassification of disease status defined by ICD codes, the date of diagnosis not reflecting the actual time of onset, and lack of information on potential covariates and unmeasured factors for HDP.

**Conclusions:**

Our findings add to the literature on associations between air pollution exposure and HDP. To our knowledge, this is the first study reporting that specific air pollution components, mixture effects, and susceptible windows of PM_2.5_ may affect GH and PE-E differently.

## Abstract 2

Yi Sun and colleagues investigate the association between hypertensive disorders in pregnancy and particulate air pollution.

## Author summary

**Why was this study done?:**

- Although previous studies have explored the associations of particulate matter ≤2.5 μm (PM_2.5_) exposure with hypertensive disorders in pregnancy (HDP), earlier findings are inconclusive.
- Several unaddressed issues remain, such as lack of simultaneous consideration of PM_2.5_ constituents, mixture effects, windows of susceptibility, and levels of disease severity.

**What did the researchers do and find?:**

- A retrospective cohort study (*N* = 386,361) was conducted using electronic health records from Kaiser Permanente Southern California (2008 to 2017) to examine the associations between HDP (mild HDP: gestational hypertension (GH) versus severe HDP: preeclampsia and eclampsia (PE-E)) and exposure to PM_2.5_, and identify windows of susceptibility to PM_2.5_ during pregnancy.
- We found higher risks for PE-E associated with exposures to PM_2.5_ total mass, organic matter, and black carbon, but not for GH. The main effect of increased PE-E risk was driven by PM_2.5_ black carbon (71%) and PM_2.5_ organic matter (26%).
- The most influential time windows of PM_2.5_ exposure associated with HDP are the late first trimester and second trimester.
- Hispanic and African American mothers and mothers who live in low- to middle-income neighborhoods may be more vulnerable to air pollution on HDP risk.

**What do these findings mean?:**

- Our findings suggest that different PM_2.5_ chemical constituents and critical exposure windows may affect GH and PE-E differently.
- Our work highlights the need for further research to investigate different components of air pollutants, levels of disease severity, and various susceptible windows, which is important to better understand the underlying mechanisms between air pollution and HDP and develop corresponding interventions.

## Introduction

Particulate matter (PM) is a complex mixture of both solid and liquid particles that spans many sizes and consists of various chemical components, including carbon and organic compounds, and inorganic compounds. The different components come from specific sources and can be grouped into primary PM, which is directly emitted from traffic, industrial processes, and burning waste; and secondary PM, which forms in the atmosphere from chemical reactions. PM with an aerodynamic diameter ≤2.5 μm (PM_2.5_) has been shown to have an adverse impact on the human body through direct deposits in the lung and, to some extent, by penetrating the alveolar membranes. These tiny particles activate inflammatory processes by releasing mediators resulting in imbalance in the autonomic nervous system and neuroendocrine pathway [1]. There is an increasing body of literature showing the adverse effects of ambient air pollution, predominantly on respiratory and cardiovascular health. Previous studies have established a strong relationship between ambient PM_2.5_ and adverse cardiovascular effects, including elevated blood pressure among the general population [2]; however, their effects on the cardiovascular system during pregnancy, including the occurrence of hypertensive disorders in pregnancy (HDP) remains unclear [3–7].

HDP, which complicates 5% to 10% of pregnancies, is a major cause of maternal and fetal morbidity and mortality [8–10]. During pregnancy, there is an increase in blood volume by 30% to 50% to adapt to the need for increased metabolic demand of the growing fetus [11]. This physiological hypervolemia during pregnancy results in an increased cardiac output. There is also a compensatory increase in heart rate in an effort to increase oxygenation in the face of dilutional hypervolemia. The concomitant increase in the rate of hyperventilation potentially leads to inhalation of more ambient toxic substances during pregnancy, which may contribute to other maternal morbidities, including HDP.

The existing epidemiological findings regarding the association of PM_2.5_ exposure with HDP are inconsistent [3–6]. Previous studies have shown both harmful impacts [12–20] and negative or null associations [21,22]. Previous studies examining the relationship between PM_2.5_ exposure and preeclampsia also reported inconsistent associations [23–28]. Inconclusive results may be partially due to differences in study populations, sample sizes, methods of exposure assessments, study regions with different PM_2.5_ levels and compositions, climate conditions, and failure to differentiate different magnitudes of HDP.

HDP are classified into 4 types based on the severity of clinical features by the American College of Obstetricians and Gynecologists (ACOG) [29]: chronic hypertension, gestational hypertension (GH), preeclampsia (PE), and chronic hypertension with superimposed preeclampsia and eclampsia (E) (S1 Appendix). The mechanisms linking air pollution and HDP subcategories may be different. For example, the pathophysiology of GH is thought to be distinct from that of PE’s in terms of alterations in placental vasculature and angiogenic factors [30]: PE-E involves chronic uteroplacental ischemia from maternal angiogenic imbalance with a changed interaction between vasoactive cytokines, leading to vasospasm [31,32], as opposed to GH. Therefore, examining HDP subcategories separately would be more elucidative about the true associations of the risk factors with HDP and the underlying mechanistic pathways [33].

Additionally, some PM_2.5_ constituents and sources may be more harmful than others to health [34,35]. Previous studies found that different PM_2.5_ constituents and exposure windows were differently associated with several adverse pregnancy outcomes, such as gestational diabetes mellitus [36] and postpartum depression [37], which is important to develop corresponding interventions targeting the main culprits and critical windows. However, very limited studies have investigated the windows of susceptibility to PM_2.5_ and HDP risk [38], and the effects of PM_2.5_ chemical compositions [12]; no prior study has explored the HDP risk related to overall mixture effects of PM_2.5_.

The objective of this study is to investigate the relationship between maternal residential exposures to PM_2.5_ total mass and its constituents (i.e., sulfate, ammonium, nitrate, organic matter, and black carbon) and HDP in a large pregnancy cohort taken from Kaiser Permanente Southern California (KPSC) electronic health record (EHR) data between 2008 and 2017. Specific aims are to (1) examine the associations between HDP and exposure to PM_2.5_ mass and its constituents by the severity of the outcomes (GH versus PE-E); and (2) identify windows of susceptibility to PM_2.5_ exposure during pregnancy. The primary hypothesis is that maternal exposure to PM_2.5_ is associated with an increased risk of developing HDP, while the associations would differ by the severity of HDP, PM_2.5_ constituents, and different exposure windows during pregnancy.

## Methods

### Study population

The participants were women with singleton pregnancies from January 1, 2008 to December 31, 2017. KPSC EHR is the source of information on demographic characteristics, residential history, individual lifestyles, medical records, and birth records. A total of 386,361 pregnancies were included in PE-E analysis after excluding participants with the following criteria (S2 Appendix): pregnancies who were not KPSC members at the time of pregnancy (i.e., mostly only having labor at KPSC and having no EHRs and outcome measures during pregnancy) or those with gestational age <20 or >43 weeks (*n* = 8,408), those with multiple fetuses (*n* = 6,694), and those without a residential address (*n* = 653). A total of 373,905 pregnancies were included in GH analysis after further excluding pregnancies complicated by chronic hypertension without superimposed preeclampsia (*n* = 12,456). Date of the last menstrual period (LMP) coupled with early pregnancy ultrasonography was utilized to determine the estimated conception date and corresponding gestational age. If there was a discrepancy between LMP and early pregnancy ultrasound report, the date found on the latter was given preference based on ACOG guidelines [39].

This study was approved by the Institutional Review Board of KPSC and the University of California, Irvine with exemption of informed consent.

### Outcome definition

Diagnoses of GH and PE-E were defined by International Classification of Diseases-9/10 (ICD-9/10) diagnostic codes [33]. Pregnant women were screened for hypertension during each prenatal visit. The HDP cases were divided into 2 groups: (1) GH: systolic blood pressure ≥140 mmHg or diastolic blood pressure ≥90 mmHg, on 2 occasions at least 4 h apart after 20 weeks of gestation in a previously normotensive woman; and (2) PE-E: with any one of the following: preeclampsia, preeclampsia superimposed upon chronic hypertension, or eclampsia (S1 Appendix).

### Exposure assessment

Monthly concentrations of PM_2.5_ total mass and its constituents (i.e., sulfate, nitrate, ammonium, organic matter, and black carbon) from 2007 to 2017 were obtained from 1-km resolution publicly available data generated by validated geoscience-derived models over North America [40,41] that included chemical transport modeling (GEOS-Chem), satellite remote sensing of aerosol optical depth, and ground-based observations combining with a geographically weighted regression. The PM_2.5_ mass estimates were consistent with ground PM_2.5_ measurements with R^2^ ranging from 0.6 to 0.85. The PM_2.5_ species in our study region of the Southwestern US has moderate to high cross-validated agreement with R^2^ values for the selected PM_2.5_ species: R^2^ sulfate = 0.59, R^2^ nitrate = 0.78, R^2^ ammonium = 0.75, R^2^ organic matter = 0.52, and R^2^ black carbon = 0.42 [40].

KPSC EHR was used to abstract information on residential histories during the entire pregnancy, including residential addresses and residency start and end dates. Monthly concentrations of PM_2.5_ total mass and components at a 1-km resolution were spatiotemporally linked to each woman based on the geocoded residential address history.

### Covariates

Covariates and potential confounders were selected from KPSC EHRs based on existing literature [3–6,42–44], including maternal age, race/ethnicity (African/American, non-Hispanic Asians, Hispanic, non-Hispanic white, and others including Pacific Islanders, Native American/Alaskan and mothers with multiple race/ethnicities specified), educational achievement (less than 8th grade, 9th grade to high school graduates, less than 4 years of college, college and more than 4 years of college); median household income at block group level in 2013; exposure to active and passive second-hand smoke during pregnancy; season of conception (warm; May–October; cool; November–April), parity (primiparous versus multiparous); pre-pregnancy body mass index (BMI, kg/m^2^, underweight: <18.5, normal weight: 18.5 to 24.9, overweight: 25.0 to 29.9, obese: ≥30.0); health insurance status (Medical or Medicare versus other kinds of health insurance) and year of infant birth. Zip Code Tabulation Areas defined by the US Census Bureau were used to represent zip codes [45]. Potential confounding factors in the relationship between air pollution and HDP were represented by a directed acyclic graph (S3 Appendix).

### Statistical analysis

Descriptive statistics were performed with distribution of selected population characteristics and PM_2.5_ exposures. We used chi-square to test the difference between pregnant women with and without GH or PE-E. Correlation between air pollution metrics was estimated with Pearson’s correlation. Multilevel Cox proportional hazard models with zip code as a random effect were applied to examine the associations between each air pollutant (PM_2.5_ total mass and 5 PM_2.5_ constituents) and each outcome during the entire pregnancy. “Entire pregnancy” was defined as the period from the conception date to the date of diagnosis for cases or the date of delivery for non-cases. Per interquartile range (IQR) increment of each air pollutant was used to calculate hazard ratios (HRs) and 95% confidence intervals (CIs). The main model was adjusted for maternal age, race/ethnicity, education, neighborhood household income, season, active and passive smoking during pregnancy, insurance type, and year of infant birth. In addition to the single-pollutant model, air pollutants with hypothesized detrimental effects on the outcome were identified and selected to be further analyzed in the multi-pollutant model. A quantile-based g-computation method from the “qgcomp” package in R was applied to measure the PM_2.5_ mixture effects. This method can estimate the effects of increasing a specific subset of exposures simultaneously controlling for possible confounding from other components in the mixture [46,47] and would be an appropriate approach to assess the joint effect of air pollution mixtures on HDP. To further identify potential windows of susceptibility during pregnancy, distributed lag models incorporating Cox models were applied to estimate the association between monthly exposure to each air pollutant and HDP risk. The lag range was defined as gestational months from the first month to the corresponding month. We considered current exposure at a given time t (month _t_), past exposure before time t (month_1_~month_t-1_, using an inverse weighting approach with weights being calculated based on time to a month _t_ to give more weight to months right before a month _t_), and potential interactions between past and current exposures (an interaction term of month _t_ × weighted month_1_~month_t-1_).

Additionally, sensitivity analyses were carried out to further examine the influence of adjusting for pre-pregnancy BMI and parity. We also applied the discrete time approach with logit function as a sensitivity analysis. The discrete-time approach is an alternative method to the Cox proportional hazard model but is more flexible without requiring the proportional hazards assumption and may be useful for handling large datasets with time-dependent variables [48,49]. The population attributable fraction (PAF) was used to estimate the proportion of cases in our population that can be attributed to PM_2.5_ exposures corresponding to the air quality standards set by the US Environmental Protection Agency (US EPA, 9 μg/m^3^) [50] and the World Health Organization (WHO, 5 μg/m^3^) [51]. Further, maternal characteristics and lifestyle behaviors such as maternal age [52], race/ethnicity [53], socioeconomic status [54], smoking [55], obesity, and primiparity [56] could be potential modifiers between air pollution and HDP. Stratified analysis was conducted to explore the differences between population subgroups. Cochran’s Q tests were used to measure the heterogeneity among subgroups. SAS version 9.4 software (SAS Institute, Cary, North Carolina, United States of America) and R 4.1.3 were used to conduct all the analyses. A two-sided *P* < 0.05 was considered statistically significant. This study is reported as per the Strengthening the Reporting of Observational Studies in Epidemiology (STROBE) guideline (S1 STROBE Checklist).

## Results

The descriptive statistics of selected demographic and pregnancy characteristics and air pollution levels are presented in Table 1. In total, 4.8% (17,977/373,905) and 5.0% (19,381/386,361) of eligible pregnancies were GH and PE-E cases, respectively. Among PE-E group, 19,334 cases of PE, 3,054 cases of preeclampsia superimposed upon chronic hypertension, and 283 cases of eclampsia were identified.

**Table 1:** Description of the study population, 2008–2017.

Characteristics	Non-HDP, *N* = 354,655	GH, *N* = 17,977	PE-E, *N* = 19,381	Total births, *N* = 386,361
Maternal age, years, mean (SD)	30.1(5.8)	30.7(5.9)	30.5(6.3)	30.3(6.1)
Maternal race/ethnicity (*N*, %)				
African American	26,554 (7.5)	1,484 (8.3)	2,197 (11.3)	29,659 (7.7)
Non-Hispanic Asian	45,061 (12.7)	1,746 (9.7)	2,097 (10.8)	48,276 (12.5)
Hispanic	181,650 (51.2)	7,872 (43.8)	10,453 (53.9)	197,108 (51.0)
Non-Hispanic white	92,449 (26.1)	6,288 (35.0)	4,099 (21.1)	101,367 (26.2)
Multiple/other	8,969 (2.5)	583 (3.2)	532 (2.7)	9,912 (2.6)
Missing	33 (0.1)	4 (0.1)	3 (0.1)	39 (0.1)
Maternal education (*N*, %)				
≤ 8th Grade	3,890 (1.1)	113 (0.6)	156 (0.8)	4,133 (1.1)
9 Grade to High School	108,911 (30.7)	5,392 (30.0)	6,070 (31.3)	118,631 (31.3)
College (<4 years)	79,696 (22.5)	4,275 (23.8)	4,923 (25.4)	87,494 (23.1)
College (4 years)	109,364 (30.8)	5,646 (31.4)	5,748 (29.7)	119,047 (31.4)
> College	45,806 (12.9)	2,221 (12.4)	2,051 (10.6)	49,437 (13.1)
Missing	6,988 (2.0)	330 (1.8)	433 (2.2)	7,639 (2.0)
Block group median household income in 2013 (*N*, %)				
<$43,667	87,920 (24.8)	4,395 (24.4)	5,721 (29.5)	96,428 (25.0)
$43,667–$55,929	88,431 (25.0)	4,457 (24.8)	4,961 (25.6)	96,493 (25.0)
$55,930–$71,591	88,303 (24.9)	4,510 (25.1)	4,688 (24.2)	96,100 (24.9)
> $71,591	88,777 (25.0)	4,562 (25.4)	3,960 (20.4)	96,023 (24.9)
Missing	1,224 (0.3)	53 (0.3)	51 (0.3)	1,317 (0.3)
Smoking (*N*, %)				
Never smoker	295,991 (83.5)	14,236 (79.2)	15,917 (82.1)	321,594 (83.2)
Ever smoker	40,175 (11.3)	2,438 (13.6)	2,332 (12.0)	44,212 (11.4)
Smoking during pregnancy	18,438 (5.2)	1,302 (7.2)	1,130 (5.8)	20,501 (5.3)
Missing	51 (0.1)	1 (0.1)	2 (0.1)	54 (0.1)
Passive smoker (*N*, %)				
Yes	7,772 (2.2)	444 (2.5)	498 (2.6)	8,572 (2.2)
No	344,644 (97.2)	17,515 (97.4)	18,827 (97.1)	375,483 (97.2)
Missing	2,239 (0.6)	18 (0.1)	56 (0.3)	2,306 (0.6)
Parity (*N*, %)				
Primiparous	141,266 (39.8)	9,604 (53.4)	10,890 (56.2)	158,534 (41.0)
Multiparous	211,283 (59.6)	8,296 (46.1)	8,360 (43.1)	225,541 (58.4)
Missing	2,106 (0.6)	77 (0.4)	131 (0.7)	2,286 (0.6)
Pre-pregnancy BMI kg/m^2^ (*N*, %)				
Underweight (<18.5)	9,239 (2.6)	156 (0.9)	282 (1.5)	9,619 (2.5)
Normal weight (18.5–24.9)	157,454 (44.4)	4,173 (23.2)	5,363 (27.7)	165,768 (42.9)
Overweight (25.0–29.9)	98,435 (27.8)	5,037 (28.0)	5,362 (27.9)	107,243 (27.8)
Obese class 1 (30.0–34.9)	50,782 (14.3)	3,777 (21.0)	4,033 (20.8)	57,348 (14.8)
Obese class 2 (35.0–39.9)	22,708 (6.4)	2,510 (14.0)	2,326 (12.0)	26,762 (6.9)
Obese class 3 (>40.0)	12,862 (3.6)	2,168 (12.1)	1,843 (9.5)	16,235 (4.2)
Missing	3,175 (0.9)	156 (1.0)	174 (0.9)	3,445 (0.9)
Insurance type (*N*, %)				
MediCal (or Medicaid)	32,224 (9.1)	1,495 (8.3)	1,917 (9.9)	35,123 (9.1)
Other	316,274 (89.2)	16,284 (90.6)	17,206 (88.8)	344,692 (89.2)
Missing	6,157 (1.7)	198 (1.4)	258 (1.3)	6,546 (1.7)
Season (*N*, %)				
Warm season (May–October)	175,113 (49.4)	9,087 (50.6)	9,407 (48.5)	190,758 (49.4)
Cool season (November–April)	179,542 (50.6)	8,890 (49.5)	9,974 (51.5)	195,603 (50.6)
Year of infant birth (*N*, %)				
2008	33,152 (9.4)	1,418 (7.9)	1,402 (7.2)	35,567 (9.2)
2009	31,614 (8.9)	1,406 (7.8)	1,455 (7.5)	34,074 (8.8)
2010	31,445 (8.9)	1,498 (8.3)	1,398 (7.2)	33,928 (8.8)
2011	33,035 (9.3)	1,751 (9.7)	1,467 (7.6)	35,795 (9.3)
2012	35,173 (9.9)	1,799 (10.0)	1,622 (8.4)	38,100 (9.9)
2013	35,738 (10.1)	1,765 (9.8)	1,820 (9.4)	38,786 (10.0)
2014	37,027 (10.4)	1,802 (10.0)	2,059 (10.6)	40,241 (10.4)
2015	38,315 (10.8)	2,039 (11.3)	2,390 (12.3)	42,045 (10.9)
2016	39,495 (11.1)	2,174 (12.1)	2,746 (14.2)	43,641 (11.3)
2017	39,661 (11.2)	2,325 (12.9)	3,022 (15.6)	44,184 (11.4)

Pregnancies complicated by chronic hypertension (*N* = 12,456) were excluded from GH analysis (*N* = 373,905).

BMI, body mass index; HDP, hypertensive disorders in pregnancy; GH, gestational hypertension; PE-E, preeclampsia-eclampsia; SD, standard deviation.

Patients with GH were more frequent among older mothers, African American or non-Hispanic white mothers, mothers with college (≤4 years), mothers who live in high-income neighborhoods, ever smokers, smoking or passive smoking mothers, mothers without MediCal/Medicaid insurance, primiparous mothers, and obese mothers; PE-E cases were more frequent among older mothers, African American or Hispanic mothers, mothers with 9 Grade to college <4 years, mothers who live in low-income neighborhoods, ever smoker, smoking or passive smoking mothers, mothers with MediCal/Medicaid insurance, primiparous mothers, and obese mothers (*p* < 0.05).

Summary statistics and Pearson correlation coefficients between air pollution metrics during the entire pregnancy are presented in Table 2. PM_2.5_ total mass was highly correlated with most PM_2.5_ chemical constituents, including PM_2.5_ organic matter (r = 0.91), nitrate (r = 0.85), black carbon (r = 0.79), and ammonium (r = 0.75), and moderately correlated with PM_2.5_ sulfate (r = 0.48). Relatively weaker correlations were noticed between sulfate and other PM_2.5_ constituents (r ≤ 0.45). Moderate to strong correlations were observed between other PM_2.5_ constituents (nitrate, ammonium, organic matter, and black carbon) (r ≥ 0.53). All the correlation coefficients in Table 2 are statistically significant (*p* < 0.05).

**Table 2:** Summary statistics and Pearson correlation coefficients between air pollution exposure metrics during the entire pregnancy.

	Mean (SD)	IQR	PM_2.5_ total mass	PM_2.5_ sulfate	PM_2.5_ nitrate	PM_2.5_ ammonium	PM_2.5_ organic matter	PM_2.5_ black carbon
**PM**_**2.5**_ **total mass**	12.86 (2.62)	3.85	1.00					
**PM**_**2.5**_ **sulfate**	1.27 (0.27)	0.35	0.48	1.00				
**PM**_**2.5**_ **nitrate**	2.41 (0.66)	0.94	0.85	0.34	1.00			
**PM**_**2.5**_ **ammonium**	0.95 (0.32)	0.40	0.75	0.45	0.80	1.00		
**PM**_**2.5**_ **organic matter**	5.39 (1.31)	1.78	0.91	0.33	0.65	0.55	1.00	
**PM**_**2.5**_ **black carbon**	1.48 (0.61)	1.05	0.79	0.23	0.54	0.53	0.72	1.00

The units for PM_2.5_ mass and PM_2.5_ constituents are μg/m^3^.

IQR, interquartile range; SD, standard deviation.

**Fig 1:**
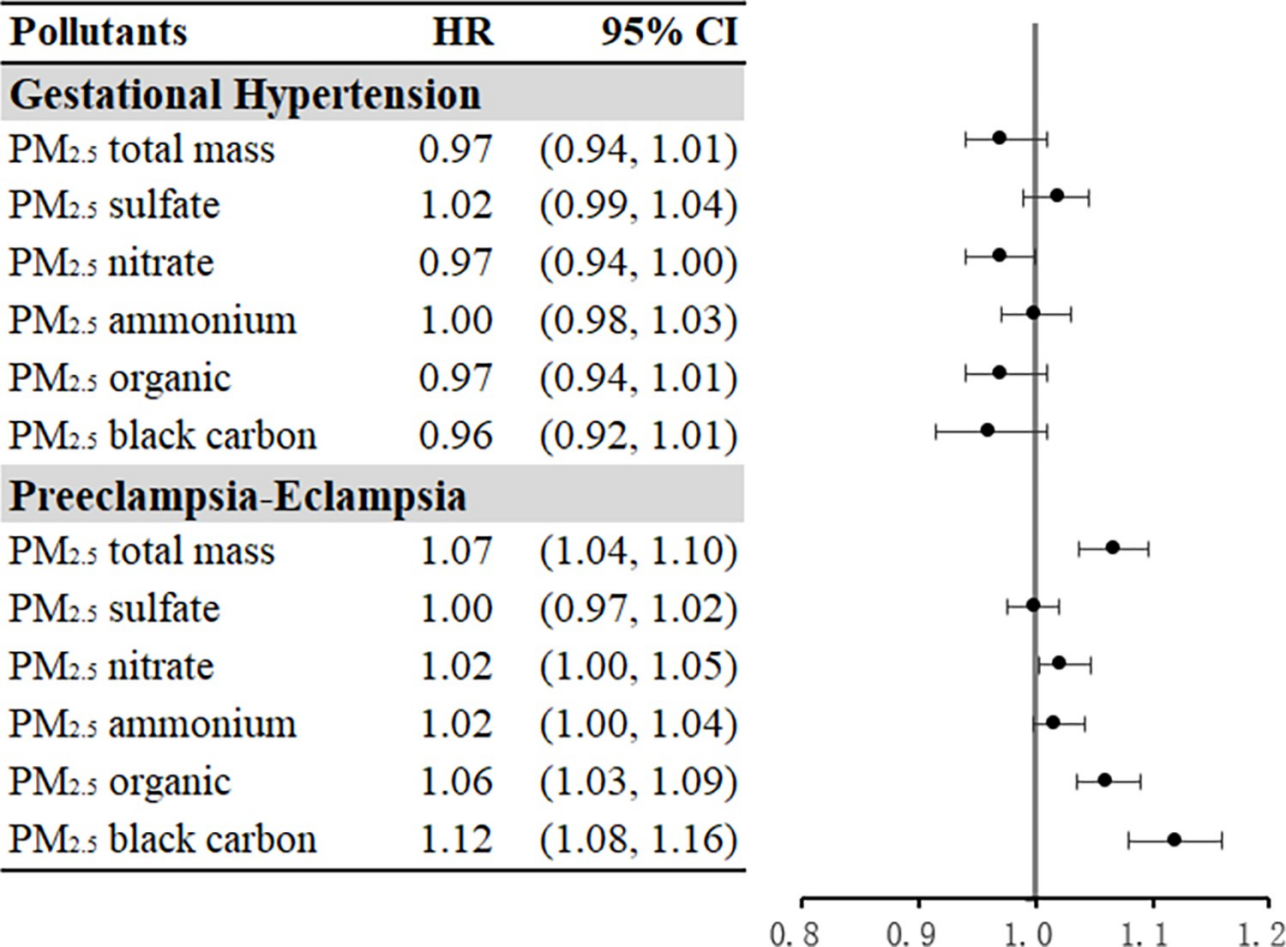
Adjusted HRs and 95% CIs of air pollution during pregnancy associated with GH and PE-E. HRs and 95% CIs were calculated for per IQR increment for each air pollutant. Model adjusted for maternal age, race/ethnicity, education, block group household income, smoking and passive smoking status during pregnancy, insurance type, season, and year of infant birth. Zip code was fitted as a random effect. CI, confidence interval; GH, gestational hypertension; HR, hazard ratio; IQR, interquartile range; PE-E, preeclampsia-eclampsia.

Results from Cox regression models are illustrated in Fig 1. No significant associations were observed between the risks of GH and PM_2.5_ and its constituents; while the relative risks of PE-E were positively associated with PM_2.5_ total mass [HR = 1.07 (95% CI [1.04, 1.10] *p* < 0.001)] and 2 PM_2.5_ constituents: highest for PM_2.5_ black carbon [HR = 1.12 (95% CI [1.08, 1.16] *p* < 0.001)], followed by PM_2.5_ organic matter [HR = 1.06 (95% CI [1.03, 1.09] *p* < 0.001)]. In sensitivity analysis (S4 Appendix), similar results were observed for associations in base models between air pollution and GH or PE-E after further adjusting for pre-pregnancy BMI and primiparity, or applying the discrete time approach models. The PAFs for PE-E corresponding to the standards of the US EPA (9 μg/m^3^) and the WHO (5 μg/m^3^) were 6.37% and 12.26%, respectively. For example, 6.37% PE-E cases among our study population could be attributed to PM_2.5_ levels above 9 μg/m^3^. In the multi-pollutant model (Table 3), β coefficients > 0 indicate positive weights of individual constituents; β coefficients < 0 indicate negative weights of individual constituents. The overall mixture coefficient ψ (log HR) from quantile g-computation is the sum of all β coefficients of the exposures of interest. Simultaneous increase in all of the 5 PM_2.5_ constituents by a quartile was associated with a higher relative risk of PE-E [HR = 1.05 (95% CI [1.03, 1.07] *p* < 0.001)], and black carbon gave the greatest contribution of overall mixture effects (70.63%) among all individual constituents, followed by organic matter (25.59%) and sulfate (3.79%).

**Table 3:** Adjusted HRs and 95% CIs of preeclampsia-eclampsia associated with 1 quartile increase in PM_2.5_ mixture during pregnancy based on quantile-based g computation.

Air pollutants	Contribution to positive/negative effect % ^c^	Positive/negative coefficient β	Overall mixture coefficient ψ (log HR) 95% CI ^d^		Overall mixture effect HR 95% CI	
**Positive mixture** ^a^						
PM_2.5_ sulfate	3.79	0.00				
PM_2.5_ organic matter	25.59	0.02				
PM_2.5_ black carbon	70.63	0.05	0.05 (0.03, 0.07)		1.05 (1.03, 1.07)	
**Negative mixture ^b^**			*P* < 0.001		*P* < 0.001	
PM_2.5_ nitrate	59.80	−0.01				
PM_2.5_ ammonium	40.20	−0.01				

HR, hazard ratio; CI, confidence interval. Model adjusted for maternal age, race/ethnicity, education, block group household income, smoking and passive smoking status during pregnancy, insurance type, season, and year of infant birth. Zip code was fitted as a random effect.

^a^The positive mixture includes pollutants positively associated with the outcome in the model.

^b^The negative mixture includes pollutants negatively associated with the outcome in the model.

^c^The sum of contribution of all positive/negative pollutants is 100.0%.

^d^The overall mixture coefficient is the sum of coefficients of the positive mixture and negative mixture.

**Fig 2:**
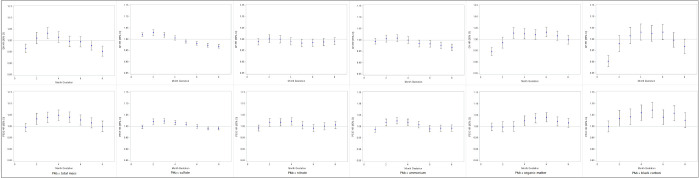
Monthly associations between maternal exposure to PM_2.5_ during pregnancy and hypertensive disorders of pregnancy. *N* = 373,905 for GH cohort; *N* = 386,361 for PE-E cohort. HRs and 95% CIs were calculated for per IQR increment for each air pollutant. Models adjusted for maternal age, race/ethnicity, education, household income, smoking and passive smoking status during pregnancy, parity, insurance type, season, and year of infant birth. CI, confidence interval; GH, gestational hypertension; HR, hazard ratio; IQR, interquartile range; PE-E, preeclampsia-eclampsia.

In the time window analyses during gestational months 1 to 8 (Fig 2 and S5 Appendix), the GH group showed negative associations with exposure to PM_2.5_ total mass at month 1 and months 7 to 8, positive associations at month 3, and nonsignificant associations for other gestational months. On the other hand, elevated risks of PE-E were associated with PM_2.5_ exposure in months 2 to 6, with the highest risk identified at month 4 [HR = 1.05 (95% CI [1.03, 1.07] *p* < 0.001)]. In terms of PM_2.5_ constituents, only exposure to PM_2.5_ sulfate (months 1 to 3) and organic matter (months 3 to 6) were associated with higher GH risks. For PE-E, windows of increased risks occurred during late first trimester and second trimester for all PM_2.5_ constituents of interest.

Subgroup analysis (S6 Appendix) was conducted stratified by maternal age, race/ethnicity, household income, smoking status during pregnancy, pre-pregnancy BMI, and primiparity. No substantial increased GH risks associated with PM_2.5_ were observed in stratified analyses. In the PE-E analyses, positive associations were significantly stronger among Hispanic and African American mothers and mothers who lived in low- to middle-income neighborhoods (*p* < 0.05), but no significant evidence of heterogeneity for other potential effect modifiers.

## Discussion

This is a large retrospective cohort study of 386,361 pregnant women residing in Southern California from 2008 to 2017. Our results show that exposures to PM_2.5_ total mass, organic matter, and black carbon were associated with an increased risk of PE-E, but not with GH. In multi-pollutant models, PM_2.5_ black carbon contributed the most to the overall mixture effects among all PM_2.5_ constituents. The late first trimester and second trimester are likely the most influential time windows for air pollution and PE-E. Furthermore, Hispanic and African American mothers and mothers who live in low- to middle-income neighborhoods were associated with higher risks for PE-E on exposure to PM_2.5_.

To the best of our knowledge, it is the first study to examine the associations between PM_2.5_ constituents and mixtures and GH and PE-E separately in a large and diverse population in regions with relatively low air pollution levels. Two recent studies in China showed positive associations between PM_2.5_ total mass and GH with null association for PE-E [12,14]. Interestingly, our study observed opposite results of positive associations between PM_2.5_ and PE-E with null associations for GH. Factors that may partially explain the inconsistent results include differences in exposure levels, exposure assessment methods, criteria for HDP diagnosis, and study region and population. For example, the PM_2.5_ concentrations in those studies were approximately 4 to 5 times (around 50 μg/m^3^) that of our study. They estimated air pollution exposure based on a hospital’s address rather than individual maternal address that may lead to more exposure misclassification. Furthermore, in those studies, the outcome was measured with physician-filled questionnaires instead of using diagnostic codes, which might introduce recall bias. Our findings of positive association of PE-E with exposure to PM_2.5_ are supported by a Swedish study with similar air pollution levels as that of our study (OR = 1.35 (95% CI [1.11, 1.63]) per 5 μg/m^3^ increment of PM_2.5_) [57]. A meta-analysis also reported that exposure to PM_2.5_ enhanced the risk of PE, but not for GH [5]. Our finding of positive associations between PE-E and PM_2.5_ black carbon is also consistent with previous studies [12,57].

Among HDP types (S1 Appendix), HDP with isolated elevated blood pressure with no systemic involvement is GH, and HDP with organ involvement or systemic manifestations (i.e., proteinuria, involvement of kidney, elevated liver enzymes, or decreased platelet count) constitute PE, that also includes superimposed preeclampsia, and hemolysis, elevated liver enzymes, and low platelet count (HELLP) syndrome, whereas, preeclampsia with neurological manifestations is considered as eclampsia. In our analyses, we observed that exposures to PM_2.5_ total mass, black carbon, and organic matter are positively associated with the severe categories in the HDP spectrum (i.e., PE-E), while the milder form of HDP (i.e., GH) may not be affected as such (S4 and S5 Appendix). The differential associations between air pollution and HDP subcategories may be explained by different mechanisms involved with mild and severe varieties of HDP. Increased expression of cytochrome P-450 and induction of stress response enzymes by PM_2.5_ have been documented [58]. The cytochrome P-450 liver enzymes induction pathway could result in rapid clearing of vaso-constrictive cytokines from the system [59]. Therefore, in cases of mild HDP, PM_2.5_ may lead to vasodilatation by inducing breakdown of vasoconstrictive factors and thus not contributing to the risk for GH. The mechanistic pathway for PE-E on the other hand has been suggested to be the result of compromised trophoblast invasion by placental vasculatures, release of placental vasoactive substances leading to marked vaso-constriction and resulting placental hypoxia [60]. PM_2.5_ exposure has been linked to endothelial dysfunction [61]. An imbalance between vasoactive substances, including vasoconstrictive factors (e.g., Thromboxane A2 and endothelin) and vasodilator factors (e.g., prostacyclin and nitric oxide), have been demonstrated as potential mechanisms of PE-E [60]. Therefore, exposure to the ambient pollution could trigger an increased production of placental vasoconstrictive factors that would overwhelm the cytochrome P-450 enzymes induction system leading to more severe categories of HDP, explaining the positive associations of pollution exposure with PE-E. Previous studies also documented elevated placental biomarker 3-nitrotyrosine and hypomethylated leptin promoters in placental tissue on exposure to PM_2.5_ and black carbon [62,63], which has been linked to high-risk pregnancies such as preeclampsia [64]. These mechanistic pathways are shown in S7 Appendix.

It should be noted that PE was more common among smoking mothers in our study population, which is different from previous studies reporting a protective effect of smoking on preeclampsia risk [65]. It has been proposed that combustion products in tobacco (e.g., carbon monoxide) [66] might be responsible for the protective effect of smoking on PE due to vasodilation. However, the inverse association between smoking and PE might be controverted due to multiple sources of bias, including eligibility criteria, losses to follow-up of women potentially at risk, misclassification, competing events, or incorrect adjustment in previous studies [55]. Further studies are warranted to extricate bias when evaluating the smoking-preeclampsia paradox.

Although several studies examined the associations between air pollution and HDP, studies explored different exposure windows are insufficient; findings are not consistent among studies, including the first, second, or third trimester [4–6]. Overall, the first and the second trimester were more susceptible to PM_2.5_ exposure in our study. Specifically, we found gestational month 3 as the critical exposure period for GH, and months 2 to 6 as the critical exposure period for PE-E. Our results are partially consistent with findings from another study in Southern California, indicating that first trimester exposure to PM_2.5_ was associated with increased odds of HDP [38]. However, this study investigated the PM_2.5_-HDP relationship among Hispanic population without considering separate HDP outcomes and had a small sample size (*n* = 298). To date, further research to identify the susceptible windows of PM_2.5_ is still needed given the inconclusive results among previous studies.

As both health and economic burdens increase with severity of HDP, more research on the associations between environmental exposure and reproductive health is needed. We used the PAF to assess the public health impact of exposure to PM_2.5_ during pregnancy. Our results showed that a 6.37% absolute reduction of PE-E cases (*N* = 1,235) in our study population would occur if PM_2.5_ exposure levels were reduced to 9 μg/m^3^ based on the US EPA air quality standards. Women with PE-E require prolonged hospitalization often including emergent cesarean section, which significantly increase health care costs. In 2003, the average (direct and indirect) PE management cost was estimated at $11,208 per woman in the United States [67]. PE has been recognized as one of the independent risk factors of future cardiovascular diseases and stroke, it also affects other vital organs, psychological health, and fetal health [68], thus contributing to the long-term social and financial impact of the health care system [69]. Although everyone is affected by air pollution, the risk may disproportionately be higher among women of certain socioeconomic backgrounds, such as women living in poverty and the minority populations in our study [70], subsequently increasing the burden of HDP. Shen and colleagues reported relatively larger effect estimates of PM_2.5_ exposure on HDP among underweight women (BMI <18.5) compared to their counterparts, but the differences between subgroups were not statistically significant [12]. Mobasher and colleagues found that PM_2.5_ exposure was significantly associated with HDP among non-obese women (BMI <30), but not obese women [38]. However, our results did not reveal any heterogeneity between the obese and non-obese subgroups. Further, identifying the main sources of air pollution associated with HDP is also important to initiate actions targeting this highly modifiable risk factor (e.g., reducing fuel emissions) to mitigate those adverse effects on reproductive health and future generations.

The main strengths of this study include the large and diverse study population in Southern California; comprehensive and detailed demographic information and medical records from KPSC EHRs allowed us to control for a number of potential confounders in our analysis; the use of time-to-event models to examine the relationship between HDP and air pollution exposure, and identify critical exposure windows; major PM_2.5_ chemical components from well-validated air pollution models; the use of an innovative statistical method to estimate the joint effects of air pollution mixtures; and consider residential changes during pregnancy, which may enhance the accuracy for the air pollution exposure assessments in this study. Furthermore, while most previous studies used combined GH and PE as the umbrella diagnosis of HDP, our study addressed the gap in the literature to link the effect of air pollution and different expressions of HDP and to understand corresponding pathophysiology.

There are certain limitations in this study. First, air pollution exposure was estimated only for residential outdoors without considering other exposure sources which could also contribute to the observed associations (e.g., indoor exposures, occupational exposures), or activity patterns due to data unavailability, which may lead to exposure misclassification and bias estimated associations in either direction. Future studies utilizing personal air pollution sampling or blood biomarkers for exposure to the pollutants would help alleviate exposure misclassification. Second, identification of disease status solely based on ICD diagnostic codes may lead to potential outcome misclassification [71]. Third, the dates of HDP diagnosis were collected from the EHR based on the routine screening during patients’ prenatal visits, which may not actually coincide with the start of the disease process. Nonetheless, the KPSC EHRs include repeated measurements from multiple time points during the entire pregnancy (7 ± 4 times) to minimize the potential bias in the estimates of time-varying exposures and outcomes. Moreover, although several covariates were adjusted, some HDP-related factors were unavailable in our analysis, such as family or personal history of HDP. In addition, further exploration of the severity and different subtypes of HDP (e.g., early-onset PE versus late-onset PE, HELLP syndrome, which is not available in our database) would give additional insights of the effects of air pollution on HDP and mechanisms. Given the complexity of air pollution and related environmental exposures, further research is also warranted to examine the effect of other air pollutants (e.g., nitrogen oxides and sulfur oxides), environmental factors (e.g., traffic noise and meteorological factors) [15,72,73], and their joint effect on HDP. Finally, animal studies examining molecular changes from air pollution exposure may shed light on the causation pathway and pathogenesis of GH and PE.

In conclusion, we found that exposures to PM_2.5_ total mass, organic matter, and black carbon were associated with an increased risk for PE-E, but not for GH. The main effect of increased PE-E risk was driven by PM_2.5_ black carbon (71%) and PM_2.5_ organic matter (26%). The susceptible window of air pollution exposure associated with PE-E is gestational months 2 to 6. Hispanic and African American mothers and mothers who live in low- to middle-income neighborhoods may be more vulnerable to air pollution on HDP risk.

## Supporting information

S1 STROBE Checklist STROBE Statement—Checklist of items that should be included in reports of *cohort studies*. (DOCX)

S1 Appendix American College of Obstetricians and Gynecologists (ACOG) classification of hypertensive disorders in pregnancy (HDP). (DOCX)

S2 Appendix Schematic flowchart for the study design with exclusion criteria. (DOCX)

S3 Appendix Directed acyclic graph (DAG) conceptualizing the relationship between variables. (DOCX)

S4 Appendix Adjusted hazard ratios (HRs) and 95% confidence intervals (CI) of GH and PE-E associated with air pollution during the entire pregnancy in sensitivity analysis. (DOCX)

S5 Appendix Monthly associations between hypertensive disorders of pregnancy and maternal exposure to PM_2.5_. (DOCX)

S6 Appendix Adjusted hazard ratios (HRs) and 95% confidence intervals (CI) of GH and PE-E associated with air pollution during the entire pregnancy among population subgroups. (DOCX)

S7 Appendix Differential pathway of pollution for GH and PE-E. (DOCX)

## Abbreviations

ACOG: American College of Obstetricians and Gynecologists
BMI: body mass index
CI: confidence interval
EHR: electronic health record
GH: gestational hypertension
HDP: hypertensive disorders in pregnancy
HELLP: hemolysis, elevated liver enzymes, and low platelet count
HR: hazard ratio
ICD-9/10: International Classification of Diseases-9/10
IQR: interquartile range
KPSC: Kaiser Permanente Southern California
LMP: last menstrual period
PAF: population attributable fraction
PE-E: preeclampsia and eclampsia
PM: particulate matter
US EPA: US Environmental Protection Agency
WHO: World Health Organization
