# Supplementary material for: Association between particulate air pollution and hypertensive disorders in pregnancy: A retrospective cohort study
Source: PLoS Med. 2024 Apr 26;21(4):e1004395. doi: 10.1371/journal.pmed.1004395 (PMC11087068; doi:10.1371/journal.pmed.1004395)
Supplement: S1 Appendix — (DOCX) [file pmed.1004395.s002.docx]

**S1 Appendix.** **American College of Obstetricians and Gynecologists (ACOG) classification of hypertensive disorders in pregnancy (HDP).**

| **Terminologies** | **Definitions** | **Prevalence, %** | **Placental vascular involvement with multisystem dysfunction**  (Braunthal & Brateanu, 2019[1]) |
| --- | --- | --- | --- |
| Gestational hypertension (GH) | SBP≥140 mmHg or a DBP ≥90 mmHg, or both, on 2 occasions at least 4 hours apart after 20 weeks of gestation in a previously normotensive woman | 5-10 (M. Shen et al., 2017[2]) | No |
| Chronic hypertension | SBP≥140 mmHg or a DBP ≥90 mmHg, or both, on 2 occasions at least 4 hours apart before 20 weeks of gestation | 3-5 (Seely & Ecker, 2014[3]) | No; general cardio-vascular involvement not specific to placental vasculatures |
| Pre-eclampsia | GH and one of the following:  Proteinuria (≥ 300 mg per 24 hours urine collection, or protein/creatinine ratio ≥0.3 mg/dL or dipstick reading ≥2 + or in the absence of proteinuria), new onset hypertension, and any of the following:  thrombocytopenia (platelet count < 100,000 x 109/L)  serum creatinine concentration >1.1 mg/dL, or doubling of serum creatinine concentrations in the absence of other diseases  elevated concentrations of liver transaminases to twice the normal concentration  pulmonary edema  new onset headache unresponsive to medication and not accounted by alternative diagnosis or visual symptoms (ACOG, 2020[4]) | 2-5 (Hutcheon et al., 2011[5]) | Yes |
| Superimposed preeclampsia | When women with chronic hypertension develop signs of preeclampsia (Banala et al., 2020[6]) | 0.2-1 (Banala et al., 2020[6]) | Yes |
| Eclampsia | HDP with convulsive manifestations | <1 (Wellington & Mulla, 2012[7]) | Yes |
| Hemolysis, Elevated Liver Enzymes and Low Platelet count (HELLP) syndrome | LDH ≥ 600 IU/dL, AST and ALT > twice the upper limit of normal levels, and platelet count < 100,000 x 109/L | 0.5-0.9 (Khalid et al., 2022[8]) | Yes |

SBP, systolic blood pressure; DBP, diastolic blood pressure; LDH, Lactate dehydrogenase; AST, Aspartate transaminase; ALT, Alanine transaminase.

**References:**

1 Braunthal, Stephanie, and Andrei Brateanu. “Hypertension in Pregnancy: Pathophysiology and Treatment.” *SAGE Open Medicine* 7 (January 2019): 205031211984370. <https://doi.org/10.1177/2050312119843700>.

2 Shen, Minxue, Graeme N. Smith, Marc Rodger, Ruth Rennicks White, Mark C. Walker, and Shi Wu Wen. “Comparison of Risk Factors and Outcomes of Gestational Hypertension and Pre-Eclampsia.” Edited by Sari Helena Räisänen. *PLOS ONE* 12, no. 4 (April 24, 2017): e0175914. <https://doi.org/10.1371/journal.pone.0175914>.

3 Seely, Ellen W., and Jeffrey Ecker. “Chronic Hypertension in Pregnancy.” *Circulation* 129, no. 11 (March 18, 2014): 1254–61. <https://doi.org/10.1161/CIRCULATIONAHA.113.003904>.

4 ACOG, 2020. Gestational Hypertension and Preeclampsia: ACOG Practice Bulletin, Number 222. *Obstetrics & Gynecology, 135*(6). <https://www.preeclampsia.org/frontend/assets/img/advocacy_resource/Gestational_Hypertension_and_Preeclampsia_ACOG_Practice_Bulletin,_Number_222_1605448006.pdf>

5 Hutcheon, Jennifer A., Sarka Lisonkova, and K.S. Joseph. “Epidemiology of Pre-Eclampsia and the Other Hypertensive Disorders of Pregnancy.” *Best Practice & Research Clinical Obstetrics & Gynaecology* 25, no. 4 (August 2011): 391–403. <https://doi.org/10.1016/j.bpobgyn.2011.01.006>.

6 Banala, Chaitra, Sindy Moreno, Yury Cruz, Rupsa C. Boelig, Gabriele Saccone, Vincenzo Berghella, Corina N. Schoen, and Amanda Roman. “Impact of the ACOG Guideline Regarding Low-Dose Aspirin for Prevention of Superimposed Preeclampsia in Women with Chronic Hypertension.” *American Journal of Obstetrics and Gynecology* 223, no. 3 (September 2020): 419.e1-419.e16. <https://doi.org/10.1016/j.ajog.2020.03.004>.

7 Wellington, K., and Z. D. Mulla. “Seasonal Trend in the Occurrence of Preeclampsia and Eclampsia in Texas.” *American Journal of Hypertension* 25, no. 1 (January 1, 2012): 115–19. <https://doi.org/10.1038/ajh.2011.173>.

8 Khalid, Farhan, Neetu Mahendraker, and Tiffany Tonismae. “HELLP Syndrome.” In *StatPearls*. Treasure Island (FL): StatPearls Publishing, 2022. <http://www.ncbi.nlm.nih.gov/books/NBK560615/>.
