# Supplementary material for: Association between particulate air pollution and hypertensive disorders in pregnancy: A retrospective cohort study
Source: PLoS Med. 2024 Apr 26;21(4):e1004395. doi: 10.1371/journal.pmed.1004395 (PMC11087068; doi:10.1371/journal.pmed.1004395)
Supplement: S2 Appendix — (DOCX) [file pmed.1004395.s003.docx]

**S2 Appendix. Schematic flowchart for the study design with exclusion criteria.**


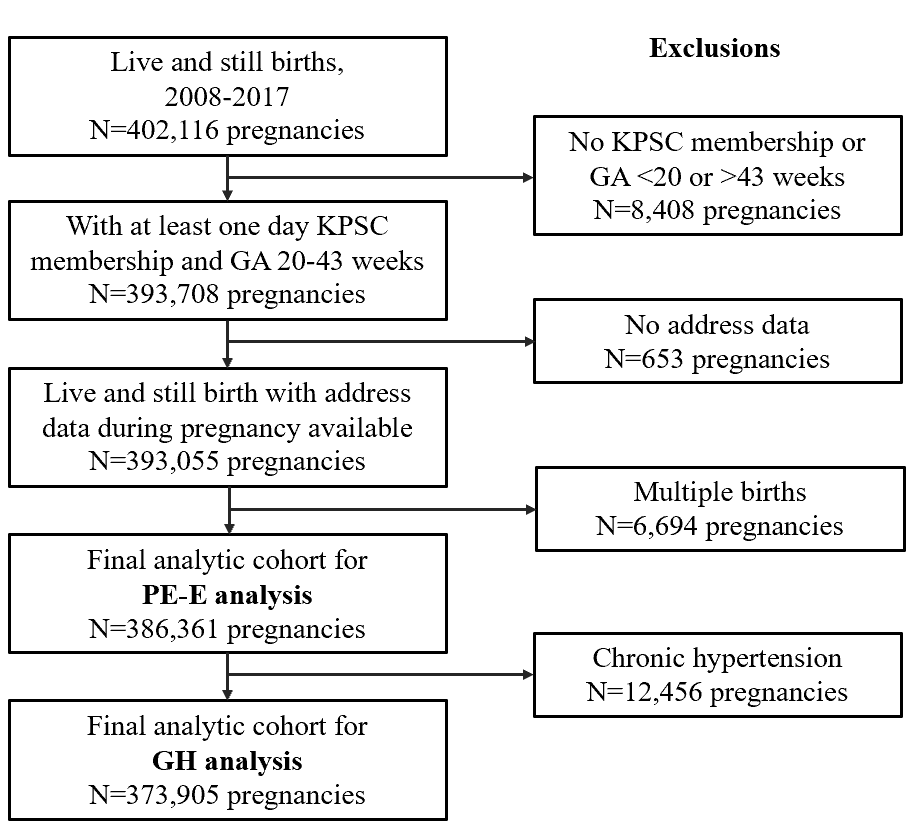


KPSC, Kaiser Permanente Southern California; GA, gestational age; GH, gestational hypertension; PE-E, preeclampsia-eclampsia (with any one of the following: preeclampsia, preeclampsia superimposed upon chronic hypertension, or eclampsia).
