# Supplementary material for: Association between particulate air pollution and hypertensive disorders in pregnancy: A retrospective cohort study
Source: PLoS Med. 2024 Apr 26;21(4):e1004395. doi: 10.1371/journal.pmed.1004395 (PMC11087068; doi:10.1371/journal.pmed.1004395)
Supplement: S3 Appendix — (DOCX) [file pmed.1004395.s004.docx]

**S3 Appendix. Directed acyclic graph (DAG) conceptualizing the relationship between variables.**


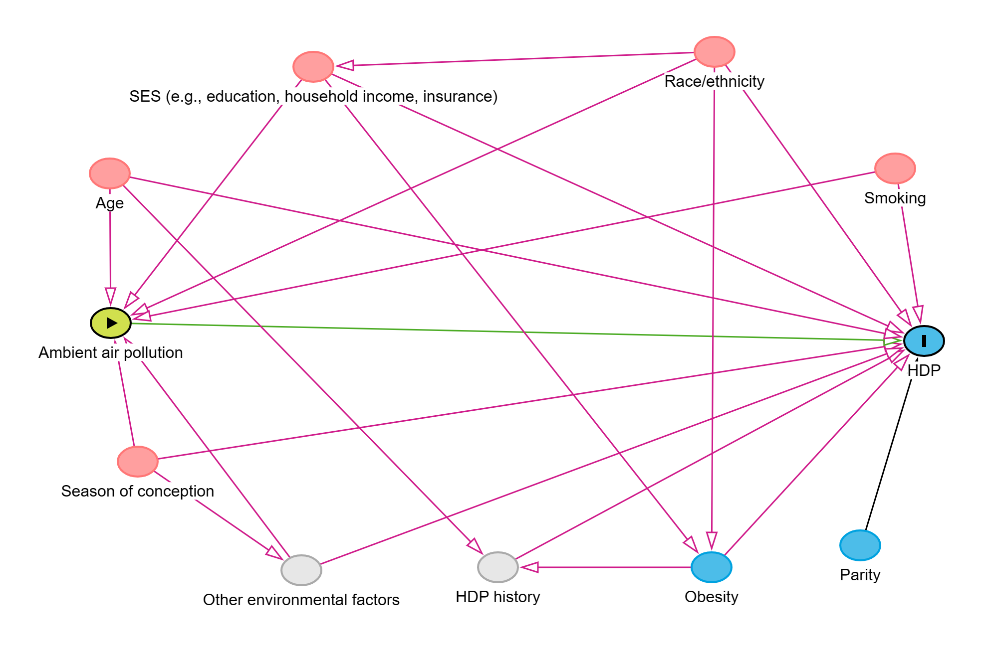


The DAG shows the relationships between air pollution exposure, hypertensive disorders in pregnancy (HDP), and other variables, where the green variable refers to the exposure, the blue variable in the black circle refers to the outcome, pink variables refer to ancestors of both the exposure and the outcome, and other blue variables refer to ancestors of the outcome (grey variables refer to unmeasured covariates). Green lines refer to the causal paths and pink lines refer to the biasing paths.
