# Supplementary material for: Association between particulate air pollution and hypertensive disorders in pregnancy: A retrospective cohort study
Source: PLoS Med. 2024 Apr 26;21(4):e1004395. doi: 10.1371/journal.pmed.1004395 (PMC11087068; doi:10.1371/journal.pmed.1004395)
Supplement: S4 Appendix — (DOCX) [file pmed.1004395.s005.docx]

**S4 Appendix. Adjusted hazard ratios (HRs) and 95% confidence intervals (CI) of GH and PE-E associated with air pollution during the entire pregnancy in sensitivity analysis.**

| **Air Pollutants** | **GH**  **HRs** | | **95% CI** | **PE-E**  **HRs** | | **95% CI** | |
| --- | --- | --- | --- | --- | --- | --- | --- |
| Base model, further adjusted for pre-pregnancy body mass index (BMI) | | | | | | | |
| PM_2.5_ total mass | 0.96 | 0.93 0.99 | | 1.05 | 1.02 | | 1.09 |
| PM_2.5_ sulfate | 0.98 | 0.96 1.00 | | 0.99 | 0.97 | | 1.01 |
| PM_2.5_ nitrate | 0.96 | 0.93 0.99 | | 1.01 | 0.98 | | 1.04 |
| PM_2.5_ ammonium | 0.96 | 0.93 1.00 | | 1.01 | 0.98 | | 1.03 |
| PM_2.5_ organic matter | 0.97 | 0.94 1.00 | | 1.05 | 1.02 | | 1.08 |
| PM_2.5_ black carbon | 0.95 | 0.91 1.00 | | 1.11 | 1.07 | | 1.15 |
| Base model, further adjusted for first pregnancy | | | | | | | |
| PM_2.5_ total mass | 0.98 | 0.94 1.01 | | 1.06 | 1.03 | | 1.09 |
| PM_2.5_ sulfate | 0.98 | 0.96 1.00 | | 1.00 | 0.98 | | 1.03 |
| PM_2.5_ nitrate | 0.96 | 0.93 1.01 | | 1.03 | 1.00 | | 1.06 |
| PM_2.5_ ammonium | 0.99 | 0.96 1.01 | | 1.01 | 0.99 | | 1.03 |
| PM_2.5_ organic matter | 0.97 | 0.94 1.00 | | 1.05 | 1.02 | | 1.08 |
| PM_2.5_ black carbon | 0.97 | 0.93 1.01 | | 1.10 | 1.05 | | 1.15 |
| Discrete-time model (Odds Ratios) | | | | | | | |
| PM_2.5_ total mass | 1.00 | 0.97 1.03 | | 1.06 | 1.04 | | 1.09 |
| PM_2.5_ sulfate | 1.00 | 0.98 1.01 | | 1.01 | 0.99 | | 1.02 |
| PM_2.5_ nitrate | 0.99 | 0.97 1.02 | | 1.02 | 1.00 | | 1.04 |
| PM_2.5_ ammonium | 1.00 | 0.98 1.03 | | 1.01 | 0.99 | | 1.03 |
| PM_2.5_ organic matter | 1.00 | 0.97 1.02 | | 1.05 | 1.03 | | 1.09 |
| 8PM_2.5_ black carbon | 1.01 | 0.97 1.05 | | 1.11 | 1.07 | | 1.16 |

GH: gestational hypertension; PE-E: preeclampsia-eclampsia. N=373,905 for GH cohort; N=386,361 for PE-E cohort. HRs and 95% CIs were calculated for per interquartile range (IQR) increment for each air pollutant. Base model adjusted for maternal age, race/ethnicity, education, household income, maternal exposure to tobacco (active and passive smoking), insurance type, season, and year of birth; zip code was fitted as a random effect.
