# Supplementary material for: Association between particulate air pollution and hypertensive disorders in pregnancy: A retrospective cohort study
Source: PLoS Med. 2024 Apr 26;21(4):e1004395. doi: 10.1371/journal.pmed.1004395 (PMC11087068; doi:10.1371/journal.pmed.1004395)
Supplement: S5 Appendix — (DOCX) [file pmed.1004395.s006.docx]

**S5 Appendix. Monthly associations between hypertensive disorders of pregnancy and maternal exposure to PM_2.5_.**

|  | **PM_2.5_ total mass** | **PM_2.5_ sulfate** | **PM_2.5_ nitrate** | **PM_2.5_ ammonium** | **PM_2.5_ organic matter** | **PM_2.5_ black carbon** |
| --- | --- | --- | --- | --- | --- | --- |
| **Gestational hypertension** | |  |  |  |  |  |
| Month 1 | **0.965 (0.946, 0.985)** | **1.020 (1.013, 1.028)** | 0.988 (0.975, 1.002) | 0.994 (0.981, 1.007) | **0.946 (0.929, 0.963)** | **0.903 (0.878, 0.928)** |
| Month 2 | 1.011 (0.986, 1.036) | **1.029 (1.017, 1.042)** | 1.002 (0.984, 1.019) | 1.006 (0.991, 1.021) | 0.985 (0.961, 1.010) | 0.980 (0.944, 1.017) |
| Month 3 | **1.032 (1.008, 1.057)** | **1.020 (1.009, 1.031)** | 1.000 (0.984, 1.017) | 1.008 (0.993, 1.023) | **1.028 (1.004, 1.052)** | 1.015 (0.979, 1.052) |
| Month 4 | 1.015 (0.991, 1.039) | 1.006 (0.996, 1.016) | 0.991 (0.974, 1.008) | 1.000 (0.985, 1.015) | **1.025 (1.002, 1.048)** | 1.031 (0.996, 1.068) |
| Month 5 | 0.997 (0.974, 1.020) | 0.991 (0.982, 1.000) | 0.983 (0.966, 1.000) | 0.984 (0.970, 1.000) | **1.021 (1.001, 1.042)** | 1.025 (0.991, 1.060) |
| Month 6 | 0.994 (0.972, 1.017) | **0.981 (0.973, 0.989)** | 0.985 (0.968, 1.001) | **0.982 (0.967, 0.996)** | **1.032 (1.011, 1.054)** | 1.031 (0.997, 1.066) |
| Month 7 | 0.975 (0.951, 1.000) | **0.972 (0.964, 0.980)** | 0.987 (0.971, 1.003) | **0.977 (0.963, 0.992)** | 1.016 (0.996, 1.037) | 0.996 (0.964, 1.030) |
| Month 8 | **0.953 (0.931, 0.975)** | **0.968 (0.960, 0.976)** | 0.991 (0.971, 1.007) | **0.967 (0.953, 0.982)** | 0.998 (0.978, 1.018) | 0.969 (0.936, 1.001) |
| **Preeclampsia-eclampsia** | |  |  |  |  |  |
| Month 1 | 0.995 (0.977, 1.014) | 0.999 (0.991, 1.006) | 0.992 (0.979, 1.006) | 0.987 (0.975, 1.000) | 0.998 (0.982, 1.015) | 1.000 (0.976, 1.024) |
| Month 2 | **1.033 (1.009, 1.057)** | **1.021 (1.009, 1.033)** | **1.017 (1.001, 1.034)** | **1.018 (1.003, 1.033)** | 0.998 (0.975, 1.021) | **1.034 (1.001, 1.070)** |
| Month 3 | **1.039 (1.016, 1.063)** | **1.023 (1.013, 1.034)** | **1.017 (1.001, 1.034)** | **1.025 (1.011, 1.040)** | 1.001 (0.979, 1.023) | **1.042 (1.008, 1.077)** |
| Month 4 | **1.049 (1.026, 1.072)** | **1.017 (1.008, 1.027)** | **1.022 (1.006, 1.038)** | **1.019 (1.004, 1.033)** | **1.026 (1.004, 1.048)** | **1.060 (1.026, 1.095)** |
| Month 5 | **1.040 (1.018, 1.063)** | **1.009 (1.001, 1.018)** | 1.004 (0.989, 1.020) | 1.009 (0.995, 1.023) | **1.037 (1.016, 1.059)** | **1.071 (1.038, 1.106)** |
| Month 6 | **1.028 (1.006, 1.051)** | 1.000 (0.992, 1.008) | 0.992 (0.976, 1.008) | 0.990 (0.977, 1.004) | **1.041 (1.021, 1.062)** | **1.040 (1.008, 1.073)** |
| Month 7 | 1.015 (0.993, 1.038) | 0.992 (0.984, 1.000) | 1.001 (0.985, 1.016) | 0.993 (0.979, 1.007) | **1.023 (1.003, 1.043)** | **1.058 (1.025, 1.091)** |
| Month 8 | 1.000 (0.978, 1.023) | 0.992 (0.983, 1.000) | 1.006 (0.990, 1.022) | 0.993 (0.979, 1.007) | 1.016 (0.996, 1.036) | 1.027 (0.994, 1.060) |

Hazards ratios (HRs) and 95% confidence intervals (CIs) were calculated for per interquartile range (IQR) increment for each air pollutant. Models adjusted for maternal age, race/ethnicity, education, household income, smoking and passive smoking status during pregnancy, insurance type, season, and year of infant birth. Bold font face indicates a statistically significant result *(P<0.05).*
