# Supplementary material for: Association between particulate air pollution and hypertensive disorders in pregnancy: A retrospective cohort study
Source: PLoS Med. 2024 Apr 26;21(4):e1004395. doi: 10.1371/journal.pmed.1004395 (PMC11087068; doi:10.1371/journal.pmed.1004395)
Supplement: S6 Appendix — (DOCX) [file pmed.1004395.s007.docx]

**S6 Appendix. Adjusted hazard ratios (HRs) and 95% confidence intervals (CI) of GH and PE-E associated with air pollution during the entire pregnancy among population subgroups.**

| **Air pollution** | **Characteristics** | **PE-E HRs** | **95%** | **CIs** | **P value for Cochran’s Q test** |
| --- | --- | --- | --- | --- | --- |
| **Maternal race/ethnicity** | |  |  |  |  |
| **PM_2.5_ sulfate** | African American | 0.99 | 0.98 | 1.06 | 0.01 |
|  | Asian | 1.04 | 0.98 | 1.10 |  |
|  | Hispanic | 1.02 | 1.01 | 1.04 |  |
|  | Non-Hispanic white | 0.95 | 0.91 | 0.99 |  |
| **PM_2.5_ black carbon** | African American | 1.11 | 1.09 | 1.23 | 0.03 |
|  | Asian | 1.06 | 1.01 | 1.15 |  |
|  | Hispanic | 1.08 | 1.01 | 1.10 |  |
|  | Non-Hispanic white | 1.08 | 1.01 | 1.13 |  |
| **Household income** | |  |  |  |  |
| **PM_2.5_ sulfate** | < $43,667 | 0.99 | 0.95 | 1.02 | 0.01 |
|  | $43,667-$55,930 | 1.05 | 1.01 | 1.08 |  |
|  | $55,930-$71,591 | 0.99 | 0.95 | 1.03 |  |
|  | >$71,591 | 0.99 | 0.96 | 1.04 |  |
| **PM_2.5_ nitrate** | < $43,667 | 1.04 | 1.00 | 1.09 | 0.04 |
|  | $43,667-$55,930 | 0.99 | 0.95 | 1.04 |  |
|  | $55,930-$71,591 | 1.08 | 1.02 | 1.13 |  |
|  | >$71,591 | 1.01 | 0.95 | 1.06 |  |
| **PM_2.5_ ammonium** | < $43,667 | 1.08 | 1.03 | 1.14 | <0.01 |
|  | $43,667-$55,930 | 1.00 | 0.96 | 1.05 |  |
|  | $55,930-$71,591 | 1.08 | 1.03 | 1.13 |  |
|  | >$71,591 | 0.99 | 0.94 | 1.04 |  |
| **PM_2.5_ black carbon** | < $43,667 | 1.20 | 1.12 | 1.28 | 0.04 |
|  | $43,667-$55,930 | 1.12 | 1.05 | 1.19 |  |
|  | $55,930-$71,591 | 1.13 | 1.06 | 1.21 |  |
|  | >$71,591 | 1.04 | 0.97 | 1.13 |  |

GH, Gestational hypertension. PE-E, Preeclampsia-Eclampsia; BMI, body mass index.

Hazards ratios (HRs) and 95% confidence intervals (CIs) were calculated for per interquartile range (IQR) increment for each air pollutant. Base model adjusted for maternal age, race/ethnicity, education, household income, maternal exposure to tobacco (active and passive smoking), insurance type, season, and year of infant birth; zip code was fitted as a random effect.
