# Supplementary material for: Association between particulate air pollution and hypertensive disorders in pregnancy: A retrospective cohort study
Source: PLoS Med. 2024 Apr 26;21(4):e1004395. doi: 10.1371/journal.pmed.1004395 (PMC11087068; doi:10.1371/journal.pmed.1004395)
Supplement: S7 Appendix — (DOCX) [file pmed.1004395.s008.docx]

**S7 Appendix. Differential pathway of pollution for GH and PE-E (Bhojwani & Agrawal, 2022).**

Cytochrome P-450 induction

Vasogenic factors going through liver for metabolism resulting in rapid metabolism of vasoconstrictors

No associated risk for GH

Release of placental vasoactive cytokines factors

Imbalance between vasoconstricting and vasodilating mediators leading to endothelial dysfunction

More risk for PE-E

**Air pollution**

Current study proposed mechanistic pathways for risks of GH versus PE-E on exposure to ambient air pollution.

GH: Gestational Hypertension; PE-E: Preeclampsia-Eclampsia.
